# Effectiveness of Treatment in General Medicine Patients With Drinking Problems

**Published:** 1994

**Authors:** David Buchsbaum

**Affiliations:** David Buchsbaum, M.D., M.S.H.A., is an associate professor in the Division of General Medicine, Virginia Commonwealth University, Richmond, Virginia

## Abstract

As many as 40 percent of patients in general medicine and family medicine practices have or have had serious alcohol-related problems. Detection of drinking problems and subsequent intervention by the primary care physician can significantly improve the outcome for these patients. Studies have shown that even brief interventions, administered during a regular office visit, can have therapeutic effects on many patients on many patients.

Alcohol is the most frequently used and abused substance in the United States. Eighty percent of adult Americans drink some alcohol (Clark and Midanik 1982), and as many as 20 percent of Americans either are experiencing alcohol-related social, legal, financial, or medical problems or are at risk for developing these problems because of their drinking (Bradley et al. 1993). Although most of these people do not seek counseling for their drinking, many will visit a general internist or family physician for routine health care in any given year. These visits provide the physician with a unique opportunity to intervene and make a difference in the patient’s life.

This article reviews the prevalence of drinking problems among patients in primary health care settings and the physician’s role in helping many of these individuals to change their drinking behaviors.

## Prevalence of Drinking Problems

**Figure 1:**
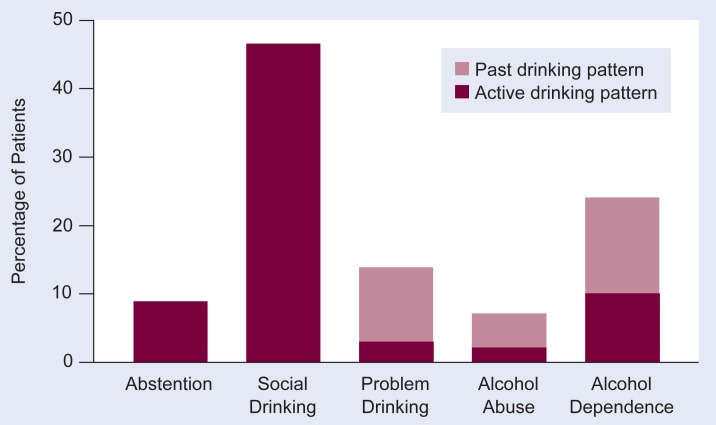
Alcohol use patterns in 453 patients attending an inner-city general medicine practice. The patients were interviewed about their drinking behavior and grouped according to the diagnostic criteria of the *Diagnostic and Statistical Manual of Mental Disorders, Third Edition* (DSM–III) into the following categories: abstention—0 drinks per week; social drinking—drinking without incurring harm by DSM–III criteria; problem drinking—meeting at least one DSM–III symptom criterion; alcohol abuse—meeting DSM–III criteria for alcohol abuse; and alcohol dependence—meeting DSM–III criteria for dependence. SOURCE: Adapted from Buchsbaum et al. 1991.

Investigations conducted in both general medicine and family medicine practice settings have shown that as many as 40 percent of patients have current or past serious problems with drinking (Cleary et al. 1988; Buchsbaum et al. 1991). Figure 1, based on a randomly chosen sample of 453 patients, demonstrates the extent of drinking problems in one inner-city general medicine practice (Buchsbaum et al. 1991). The data were collected through formal interviews according to the Diagnostic Interview Schedule (DIS), a diagnostic instrument that is based on the *Diagnostic and Statistical Manual of Mental Disorders, Third Edition* (DSM–III; American Psychiatric Association 1980). The DSM–III diagnostic criteria for alcohol abuse or dependence highlight the behavioral, medical, and social consequences of excessive or uncontrolled drinking. In the sample, 12 percent of the patients met the diagnostic criteria for active alcohol dependence or abuse, and 3 percent, although “subthreshold” for a formal DSM–III diagnosis, were harming themselves by their pattern of drinking and were classified as “problem drinkers.” An additional 30 percent of the patients were identified as previous problem, alcohol-abusing, or alcohol-dependent drinkers.

In addition to these patients with serious alcohol-related problems, an equal or higher number of patients are drinking in a manner that places them at risk for experiencing adverse medical or social complications such as liver disease or marital discord. These patients also could benefit from a physician’s attention to their drinking habits. To identify them as well as those patients with obvious signs of alcohol abuse, the physician can consider using three diagnostic categories: hazardous drinking, harmful drinking, and dependent drinking.

Hazardous drinking (also sometimes called at-risk drinking) refers to a pattern or level of drinking that, if continued, puts the patient at risk for adverse social, medical, or psychological consequences (Babor and Grant 1992). This category includes people who drink more than 14 drinks^1^ per week or more than 5 drinks per drinking session (Bradley et al. 1993). Many of them are not aware that their drinking represents a hazard. Harmful drinking is a category based on the World Health Organization’s *International Classification of Diseases*. It is defined as a drinking behavior that has already resulted in adverse consequences. This definition shares diagnostic elements with the alcohol abuse diagnosis as defined in the DSM scheme. Finally, dependent drinking refers to a pattern of drinking that is characterized by symptoms of withdrawal upon discontinuation. This term refers to a diagnosis based on DSM criteria.

The relative proportions of these disorders in a given medical practice depend on the location of the practice and its patient composition. In settings characterized by high levels of economic indigence such as inner cities, alcohol dependence may be more common than harmful and hazardous drinking. In non-inner-city primary care populations, on the other hand, hazardous and harmful drinking combined are more common than dependent drinking (Skinner 1990). Further, all drinking disorders are three times more common in male patients than in female and more common in patients 50 years or younger (Buchsbaum et al. 1991). With a minimal investment of time and effort, a primary care physician can influence many of these patients to alter their drinking behaviors. But the physician must first recognize that a problem exists.

## Physician Detection of Drinking Problems

Detection of a drinking problem is the single most important determinant of physician intervention. In a study conducted in a large Boston clinic, Cleary and coworkers (1988) found that physicians detected an alcohol abuse problem only in less than half of the patients afflicted. The physicians then counseled or offered treatment to approximately two-thirds of the patients they identified as having a current alcohol problem.

Several factors appear to influence physician awareness and detection of drinking problems. First, many physicians underestimate the prevalence of alcohol abuse disorders in their patients (Linn and Yager 1989). This is important to detection, because the higher the prevalence of a problem, the more likely the physician will consider the diagnosis when evaluating a patient’s complaints (Elstein et al. 1978). Consistent with this notion, physicians detect fewer alcohol problems in female patients, who have a lower prevalence of alcohol disorders than men (Bucholz 1992; Buchsbaum et al. 1992*a*).

Second, some physicians may have attitudes, knowledge, and beliefs that in effect limit their efficiency in detecting and treating drinking problems (Geller et al. 1989). Most physicians have only limited exposure to a formal substance abuse curriculum during their medical school and postgraduate medical training. Consequently, their opinions and expectations are shaped mainly by their experience with hospitalized “alcoholics” who have been admitted for the most dire consequences of drinking and who are the most difficult to treat. This experience with extreme cases of dependence may reinforce the physicians’ belief that their treatment efforts will go unrewarded (Johnson and Clark 1989).

Furthermore, physicians tend to rely more on evidence of stereotypical and advanced complications of alcoholism, such as advanced liver or pancreatic disease, than on gathering information on the patient’s drinking behaviors (Buchsbaum et al. 1992*a*). As a result, physicians are more likely to identify seriously impaired drinkers who are least likely to respond to simple treatment measures (Buchsbaum et al. 1992*a*). This could explain why many physicians do not consider the treatment of patients with drinking problems to be within the scope of their therapeutic role (Wechsler et al. 1983).

## Enhancing Detection

Because of these limitations in the detection of alcohol-related problems in primary care patients, more attention has been directed at ways to improve rates of detection through systematic screening in the physician’s practice. Several screening questionnaires have been developed and advocated for use in the primary care setting, including the CAGE test (acronym for Cut down, Annoyed, Guilty, and Eye opener); the MAST (Michigan Alcoholism Screening Test) and its many derivatives; and, more recently, the AUDIT (Alcohol Use Disorders Identification Test)(Ewing 1984; Selzer 1971; Saunders et al. 1993; for more information on screening instruments, see Nilssen and Cone, pp. 136–139).

These questionnaires identify the symptoms of physical, psychological, or social disruption that are common to harmful or dependent drinking. Their screening performance in identifying hazardous/harmful and dependent drinkers is summarized in table 1. The data were compiled from studies reported in the literature (Bernadt et al. 1982; Fleming et al. 1991; Peterson et al. 1983; Buchsbaum et al. 1992*b*; Magruder-Habib et al. 1993). The table shows that the three questionnaire-based screening tests perform comparably and, if used systematically, could substantially increase the number of patients who ultimately receive attention for their drinking behavior.

The CAGE test, because of its brevity, probably has the greatest clinical use, but it cannot distinguish between a current and a past drinking problem. The AUDIT, on the other hand, specifically assesses the drinking activity during the past year and further characterizes drinking patterns by including questions related to the quantity and frequency of drinking. It is more difficult to score and takes longer to administer than the CAGE test, making it somewhat less attractive to the busy clinician.

The MAST also has drawbacks as a screening tool: it does not establish a timeframe for the drinking problem, and it takes longer to administer than the CAGE test. An advantage is that scores on the MAST, as on the AUDIT, can be used to group patients according to problem severity, because higher scores correlate with greater impairment.

When administered routinely, these screening instruments can effectively identify patients with drinking problems. Yet it is currently unknown whether physicians will use them systematically if trained to do so. To make use of the screening tests less dependent on physician cooperation, they can be self-administered by the patients while they are awaiting their visit or administered by nonphysician personnel. The results can then be given to the physician. Providing physicians with such information can increase their rates of intervention by 50 percent (Buchsbaum et al. 1993).

Tests for biological markers, such as the alcohol-specific liver enzyme gamma-glutamyl transferase (GGT), or a combination of biological markers also may be helpful in identifying harmful, hazardous, and dependent drinkers. However, because of their low sensitivity (table 1), biological markers such as the GGT test generally are not recommended as exclusive screening measures (Bernadt et al. 1982; Cushman et al. 1984; Peterson et al. 1983; for more information on biological markers of alcohol abuse, see the article by Salaspuro, pp. 131–135).

## Brief Interventions

Primary care physicians can alter treatment outcomes in many patients with drinking problems. The time demands of a busy practice, however, and lack of developed counseling skills may discourage a physician’s effort to initiate treatment. Because a physician is more likely to conduct an intervention that is quick, simple to administer, and effective, several minimal interventions have been developed.

These treatment options, which can be provided by the physician during a 5- to 15-minute office visit, include simple directives (advice), reading materials such as self-help manuals (bibliotherapy), and referral to a treatment specialist or treatment program. This last approach is particularly important to the treatment of alcohol-dependent drinkers or severely impaired harmful drinkers. The physician’s role here is to confront the patient, advise the patient that his or her pattern of drinking is unhealthy, and prepare the patient for possible referral. Compliance with the referral appointment has been shown to be enhanced if the patient is referred immediately to a health professional practicing at the same site (Goldberg et al. 1991).

As will be discussed subsequently, providing advice and reading materials is especially appropriate for drinkers who fall into the categories of hazardous drinking and less seriously impaired harmful drinking. In its simplest form, advice entails assessing the extent of alcohol-related impairment, discussing the consequences of continued hazardous drinking, and advising the drinker to either cut down or abstain. These directives may be further enhanced by providing the patient with reinforcing reading material (Heather et al. 1987).

### Brief Interventions in Inpatient Settings

The notion that physicians could facilitate changes in the patient’s drinking behavior through simple advice is supported by studies first conducted in inpatient medical settings. In an early study of brief interventions, Edwards and colleagues (1977) found that participation in a session of structured advice was as effective in changing drinking behaviors of self-referred alcoholics, as was a more conventional and intensive inpatient treatment program followed by outpatient treatment sessions. It is important to note that because these individuals were self-referred, their motivation for treatment may have been higher than can be expected of a patient seeing a physician for a non-alcohol-related problem. Nonetheless, the results of this investigation did support the premise that brief advice alone may have been effective in these patients.

In another study of the usefulness of brief interventions for hospitalized men, Chick and colleagues (1985) found that exposure to a 10-minute health behavior screening interview that included questions about alcohol use and alcohol-related problems could have profound therapeutic effects on drinking behaviors. In this study, all patients who screened positive on a problem drinking inventory were assigned to either receive a counseling session of up to 1 hour prior to discharge or receive neither counseling nor any discussion about their screening interview.

After 1 year, patients in both groups reported similar 50 percent declines in alcohol consumption. Although the additional hour of counseling did not appear to have an appreciable effect in reducing alcohol consumption, counseled patients did report significantly fewer drinking-related problems than noncounseled patients did. It is difficult to estimate how much improvement was due to the passage of time alone because no controls were used.

These studies on inpatients set the stage for evaluating the effect of advice on the behaviors of hazardous or harmful drinkers identified in outpatient settings. For a complete review of these outpatient studies, the reader is referred to an article by Bien and colleagues (1993). Three of the largest and best designed of these investigations are discussed below.

### Brief Interventions in Outpatient Settings

In the first study, Wallace and colleagues (1988) followed more than 900 British general medicine patients who drank excessively and had been assigned randomly to either a treatment or a control group. “Excessive” was defined as drinking at least the equivalent of 54 standard drinks per week for men and 31 drinks for women. The treatment group subjects were given feedback on their drinking, advised to cut down, asked to maintain a drinking diary, and given a self-help booklet by their physician. The control subjects received no treatment.

After 6 months, male patients receiving treatment reported a 25-percent decline in their weekly consumption versus 13 percent in the control males. These differences remained the same after 1 year of treatment. Subsequent analyses revealed that the number of treatment sessions during the year correlated positively with improvement. Female patients also showed substantial declines in their reported consumption 6 months later, but the differences between the treatment and control groups were not statistically significant. After 1 year, however, the differences (33 percent decline in the treated females, 17 percent decline in the control females) did achieve significance and were comparable with those of male patients.

In the second study, another randomized trial of brief intervention, Anderson and Scott (1992) tested the effect of an advice-based brief intervention on 154 male patients who, on a self-administered Health Survey Questionnaire, reported drinking the equivalent of 30 standard drinks or more per week. Based on their alcohol-related problems at entry, the majority of these patients were classified as “harmful” drinkers. Subjects were assigned randomly to either a brief intervention group or a control group whose members only completed the questionnaire. Brief intervention included 10 minutes of advice, a self-help booklet, and feedback concerning the results of tests for the biological marker GGT. After 1 year, 100 of the subjects, equally distributed between the intervention and control groups, were reexamined. Subjects in the intervention group reported a 30-percent reduction in their weekly alcohol consumption, whereas the subjects in the control group reported an 18-percent reduction.

Scott and Anderson (1991) also performed a companion study on the effectiveness of advice in females who reported drinking the equivalent of 31 or more drinks per week. Both the advised subjects and the control subjects, who received only the screening interview, reported a 42-percent reduction in consumption after 1 year. Advice therefore did not seem to confer an additional treatment effect.

Finally, in the largest study of brief intervention in primary health care settings to date, Babor and Grant (1992) conducted a multinational study involving more than 1,600 male and female harmful drinkers as defined by their scores on the AUDIT screening instrument. In the design of this project, participants were identified through a 20-minute health interview and then randomly assigned to one of three groups. The first group received 5 minutes of advice; the second group received advice, 15 minutes of counseling plus a self-help manual; and the third group received no treatment other than that imparted by screening. Six months later, the following effects were found: advice-only patients reported a 38-percent reduction in daily consumption, counseled patients reported a 32-percent reduction, and control patients reported a 10-percent reduction. Notably, counseling conferred no additional benefit over advice-only treatment. In this study, a treatment effect was observed only in male patients. The drinking behavior of female patients improved with and without treatment.

Of these three primary care-based studies, only Babor and Grant’s design excluded dependent drinkers. The effect of brief interventions on the behaviors of dependent drinkers is not as clear.

## Future Questions

Multiple studies conducted in both inpatient and outpatient health care settings have shown that physicians can improve the treatment outcomes of many of their patients with drinking problems. For the hazardous and harmful drinker, this may result directly from the physician’s detection and intervention efforts. For the dependent drinker, the physician can be the critical first link in a treatment chain that begins with detection and referral to treatment specialists.

The data discussed in this article raise intriguing questions that when answered will improve the treatment of patients with alcohol problems in the primary care setting. First, how intense an intervention is necessary to induce a change in drinking behaviors? In contrast to conventional treatment options that rely on the drinkers’ awareness of need, the initiation of treatment in general medicine and other primary care settings is most often the result of an opportunistic encounter where the patients have not sought help specifically for their drinking. In this situation, the patients’ readiness to change their drinking behavior may be an important factor in determining their responsiveness to intervention (DiClemente and Hughes 1990). Future studies that match patients to different options and intensities of treatment will provide information on the degree of intensity needed to change behavior.

Second, how does patient gender relate to the intensity of treatment needed? In several studies mentioned above, treatment effects were most pronounced in male patients. In female patients, the screening interview itself appeared to confer a therapeutic effect that was improved only marginally by counseling or advice. This suggests that additional effects beyond those derived from screening may require more intensive treatment in female patients.

Third, what is the effect of time on drinking behavior? In all of the studies reviewed in this article, drinking levels declined over the period of observation regardless of intervention status. This suggests that drinking behaviors are dynamic and that diagnostic categories are unstable with respect to time. Along these lines, studies show that twice as many of the general medicine patients reported past drinking problems rather than current problems (Buchsbaum et al. 1991). Interestingly, the large majority of these drinkers never underwent formal treatment. This phenomenon of spontaneous remission and the factors important to its occurrence must be examined and accounted for in future studies.

Fourth, how does the patients’ social environment influence the success of brief treatment modalities? An intervention that presumes the existence of a supportive environment characterized by the presence of a spouse or spouse equivalent and a stable home and work history, may be less effective in a population of patients who lack such supportive environmental elements.

Finally, given the number of potential treatment candidates, how can the primary care setting be organized to best accommodate the short- and long-term treatment needs of the drinking population? Although most of the published literature promotes the central role of the physician in brief interventions, other allied health personnel also can play significant roles. The organization of health care personnel into functional treatment teams composed of physicians, nurses, social workers, and technicians warrants particular attention.

## Figures and Tables

**Table 1:** Screening Performance of Four Tests Used To Identify Hazardous/Harmful and Dependent Drinkers—the CAGE, MAST, AUDIT, and GGT

Test	Cutoff Score^1^/Maximum Score	Hazardous/Harmful Drinkers		Dependent Drinkers	
	
		Sensitivity^2^ %	Specificity^3^ %	Sensitivity %	Specificity %
CAGE	≥ 2/4	50	92	74	91
MAST	≥ 5/53	85	87	84	87
AUDIT	> 8/40	92	94	68	85
GGT	> 40 IU/L^4^	< 50	65	< 50	85

1 The cutoff score approximates a balance between sensitivity and specificity. Scores above the cutoff are regarded as positive and those that fall below as negative. For example, a “yes” response to two of the four CAGE questions or a score of 5 out of 53 points on the MAST indicate alcohol abuse and suggest further assessment.

2 Sensitivity is a test’s ability to detect disease in a patient with the disease.

3 Specificity represents a test’s ability to rule out disease in a patient who does not have the disease.

4 The GGT is a biological test that measures the level of the liver enzyme gamma-glutamyl transferase (GGT) in the blood serum. A result of 40 international units per liter (IU/L) and higher is suggestive of long-term excessive alcohol consumption.

## Footnotes

1 One standard drink is defined as 12 fluid ounces of beer, 5 fluid ounces of wine, or 1.5 fluid ounces of distilled spirits.
